# Resveratrol Attenuates High Glucose-Induced Osteoblast Dysfunction *via* AKT/GSK3β/FYN-Mediated NRF2 Activation

**DOI:** 10.3389/fphar.2022.862618

**Published:** 2022-05-23

**Authors:** Yue Xuan, Jie Wang, Xiaohui Zhang, Jie Wang, Jiahao Li, Qingbo Liu, Guangping Lu, Mengjie Xiao, Ting Gao, Yuanfang Guo, Cong Cao, Ou Chen, Kunli Wang, Yufeng Tang, Junlian Gu

**Affiliations:** ^1^ School of Nursing and Rehabilitation, Cheeloo College of Medicine, Shandong University, Jinan, China; ^2^ Department of Orthopedic Surgery, The First Affiliated Hospital of Shandong First Medical University, Jinan, China

**Keywords:** resveratrol, diabetes, osteoblast dysfunction, oxidative stress, NRF2

## Abstract

Osteoblast dysfunction, induced by high glucose (HG), negatively impacts bone homeostasis and contributes to the pathology of diabetic osteoporosis (DOP). One of the most widely recognized mechanisms for osteoblast dysfunction is oxidative stress. Resveratrol (RES) is a bioactive antioxidant compound to combat oxidative damage. However, its role in the protection of HG-induced osteoblast dysfunction has not been clarified. Therefore, our study aimed to explore potential regulatory mechanisms of RES for attenuating HG-induced osteoblast dysfunction. Our results showed that osteoblast dysfunction under HG condition was significantly ameliorated by RES *via* the activation of nuclear factor erythroid 2-related factor (NRF2) to suppress oxidative stress. Furthermore, using *Nrf2*-shRNA and wortmannin, we identified that activation of NRF2 *via* RES was regulated by the AKT/glycogen synthase kinase 3β (GSK3β)/FYN axis.

## Introduction

Diabetic osteoporosis (DOP) is a chronic bone metabolic disease caused by diabetes mellitus (Mohsin et al., 2019). Studies showed that diabetes may contribute to the loss of bone density and deterioration of the bone structure, eventually resulting in a high risk of bone fracture and disability (Giangregorio et al., 2012; Fonseca et al., 2014). Notably, the incidence of hip fractures is about six-time higher in type 1 diabetes patients than non-diabetic population (Schwartz 2017). However, there is no definitive treatment to prevent or reverse DOP to date. Moreover, precise mechanisms for DOP have not been fully clarified. Bisphosphonates, one of the most common and long-term anti-osteoporosis drugs, reduce the risks of bone fractures in postmenopausal women with diabetes (Khosla and Hofbauer, 2017; Mohsin et al., 2019). Unfortunately, Burr et al. found that dogs treated with alendronate (a bisphosphonate) at a clinical dose exhibited increasingly brittle behavior in a time-dependent manner, demonstrating that bisphosphonates might not be proper for long-duration treatment (Burr et al., 2015). Thus, it is necessary to explore therapeutic interventions aimed at the improvement of DOP. Accumulating evidence demonstrates that oxidative stress is a crucial determinant of osteoporosis (Fatokun et al., 2006; Callaway and Jiang, 2015). Oxidative stress is defined as an imbalance of the redox state in cells, with the production of excessive reactive oxygen species (ROS) and insufficient antioxidants (Sanchez-Rodriguez et al., 2007). Delaying the process of osteoporosis could be achieved by the suppression of oxidative stress (Zhu et al., 2018). Hamada et al. also revealed that increased oxidative stress levels could be detected in bone tissues of diabetic mice (Hamada et al., 2009). These findings support the great importance of oxidative stress in the development of osteoporosis, especially in the setting of diabetes, and strategies to reduce ROS levels would become promising therapies for DOP.

Osteoblasts play a fundamental role in maintaining bone quality and function (Li et al., 2020). Osteoblast dysfunction, such as increased oxidative stress, surely damages normal bone structure and homeostasis (Callaway and Jiang 2015). Therefore, activating the endogenous antioxidant system may become a feasible approach to preserving bone health for diabetic patients. Nuclear factor erythroid 2-related factor 2 (NRF2), well-known as a redox-sensitive transcription factor, contributes to the defense system against oxidative damage in diabetic prevention and care *via* activation of antioxidant genes (Li et al., 2012). The essential role of NRF2 in the maintenance of osteoblast function has been widely appreciated. Zhao Z. et al. (2021) found that activin receptor-like kinase 7 silencing activated NRF2 to protect osteoblasts against high glucose- (HG-) increased oxidative stress. This indicates that NRF2 activation is strongly associated with inhibition of oxidative stress induced by HG in the treatment of osteoblast dysfunction. Hence, finding out potential therapies that can activate NRF2 under the HG condition becomes a breakthrough in clinical treatments for DOP. Resveratrol (RES), a natural polyphenol derived from fruits and nuts, possesses a powerful antioxidant capacity by promoting the nuclear localization of NRF2 (Farkhondeh et al., 2020). Wang et al. showed that RES ameliorated diabetic cardiomyopathy by targeting NRF2 activity (Wang et al., 2018). However, whether RES exerts protective effects on osteoblast dysfunction following HG damage remains to be determined. RES was reported to activate autophagy *via* the AKT signaling pathway to improve cognitive dysfunction with chronic cerebral hypoperfusion (Wang et al., 2019). Furthermore, the activity of NRF2 could be regulated by AKT/glycogen synthase kinase 3β (GSK3β)/FYN signaling pathway in stromal cell-derived factor 1-treated endothelial progenitor cells by controlling FYN-mediated export and degradation of nuclear NRF2 (Dai et al., 2017). Based on the above findings, our study aims to explore 1) the protective effects of RES on osteoblast dysfunction following HG damage and 2) whether RES rescues osteoblast dysfunction from HG damage through activating NRF2 *via* AKT/GSK3β/FYN pathway.

## Materials and Methods

### Cell Culture

MC3T3-E1 cells (a mouse preosteoblast cell line), purchased from American Type Culture Collection (ATCC, VA, United States), were cultured in α-minimum essential medium (α-MEM, 5.6 mM D-glucose, Servicebio technology, Wuhan, China) containing 10% fetal bovine serum (Gibco, Grand Island, NY, United States) and 1% antibiotics (10,000 U/ml penicillin and 10 mg/ml streptomycin, Gibco) at 37°C with 5% CO_2_. To induce osteogenic differentiation, a basic culture medium, adding β-glycerophosphate (10 mM, Yuanye Biotechnology, Shanghai, China) and ascorbic acid (50 mg/ml, Solarbio Life Sciences, Beijing, China), was used to culture cells (Zhao L. et al., 2021). The osteogenic differentiation medium was replaced every 3 days.

### Cell Treatment

Cells were treated with vehicle, high glucose (30 mM, Sigma-Aldrich, St. Louis, MO, United States) based on previous studies or RES (5 μM, MedChemExpress, NJ, United States) in a basic culture medium for 24 h or osteogenic differentiation medium for 14 days. When using wortmannin (MedChemExpress) as an inhibitor of phosphatidylinositol 3-kinase (PI3K)/AKT, cells were pretreated with 10 μM wortmannin for 6 h and then subjected to indicated treatments for another 24 h before conducting experiments.

### Cell Transfection

Cells were transfected with negative control-short hairpin RNA (NC-shRNA) or *Nrf2*-shRNA (GenePharma, Shanghai, China) *via* cell transfection reagent (Obio Technology, Shanghai, China). Procedures were conducted by a standard protocol based on the manufacturer’s instructions.

### Cell Viability

Cell viability was performed by Cell Counting Kit 8 (CCK8, Beyotime Biotechnology, Shanghai, China). Cells were planted in 96-well plates, then treated with or without HG and RES (0.1, 1, and 5 μM) for 24 h, and finally measured. In order to ensure the effective time of RES, cells were treated with HG or HG combined with RES (5 μM) for 12, 24, or 48 h. After indicated treatments, 10 μl CCK8 reagent was added to each well and incubated at 37°C before detecting OD values at 450 nm. Cell viability was calculated using OD values and expressed by percentage.

### Cell Staining

In order to detect cell apoptosis, an *In Situ* Cell Death Detection Kit (Sigma-Aldrich) was used, and nuclei were counterstained with DAPI (Abcam, Cambridge, United Kingdom). For evaluating the ability of osteogenic differentiation, alkaline phosphatase (ALP) staining and alizarin red staining (ARS) were carried out by using a BCIP/NBT ALP color development kit (Beyotime Biotechnology) and Alizarin Red S (Solarbio Life Sciences), respectively. Measuring ROS levels, cells were stained with a dihydroethidium (DHE) fluorescence kit (Beyotime Biotechnology) and 2’,7’-dichlorofluorescin diacetate (DCFH-DA) fluorescence kit (Beyotime Biotechnology). For immunofluorescent staining, primary antibodies, anti-FYN antibody (1:500, Cell Signaling Technology, Danvers, MA, United States), and anti-NRF2 antibody (1:200, Abcam) were used, respectively. The secondary antibody was an anti-Rabbit IgG H and L Alexa Fluor 647 (1:200, Abcam). DAPI was applied to stain nuclei. All above staining was conducted according to the manufacturers’ instructions. Staining results were observed and photographed by light microscope (Nikon, Tokyo, Japan) or fluorescence microscope (Nikon) and calculated by ImageJ software.

### ALP Activity Assay

Cells were cultured in an osteogenic differentiation medium for 7 days with indicated treatments, harvested, and prepared for the detection of ALP activity by the ALP activity assay kit (Beyotime Biotechnology) following the standard protocol. The absorbance was measured at 405 nm.

### Catalase and Superoxide Dismutase Activities Assay

Cells were seeded in six-well culture plates and treated with HG or RES or both for 24 h to measure the activities of antioxidant enzymes (CAT and SOD). Then, cell lysates were collected and measured by CAT and SOD Assay Kit (Solarbio Life Sciences), respectively, following the manufacturer’s instructions.

### Real-Time Quantitative PCR

Total RNA was extracted from cells using TRIzol reagent (Cwbio, Valencia, CA, United States). Then, 1 μg of total RNA was used for reverse transcription into cDNA by HiFiScript cDNA Synthesis Kit (Cwbio). Finally, cDNA was amplified by a real-time PCR detection system (Bio-Rad, Hercules, CA, United States) with UltraSYBR Mixture (Cwbio) using the following gene-specific primers (Sangon Biotech, Shanghai, China): heme oxygenase 1 (*Ho1*), NAD(P)H quinone dehydrogenase 1 (*Nqo1*), *Cat*, *Sod*, osterix (*Osx*), collagen 1 (*Col1*), osteopontin (*Opn*), runt-related transcription factor 2 (*Runx2*), and glyceraldehyde-3-phosphate dehydrogenase (*Gapdh*). PCR was performed according to the manufacturer’s instructions*.*

### Western Blot Assay

Proteins were extracted from cells *via* radioimmunoprecipitation assay buffer (Beyotime Biotechnology) after different treatments, then equally separated by 10% SDS-PAGE gel, and ultimately transferred into nitrocellulose membranes (GE Healthcare Life Sciences, Beijing, China). Membranes were blocked in 5% nonfat milk for 1 h, and incubated overnight at 4°C with anti-phosphorylated-AKT (P-AKT, 1:1,000, Cell Signaling Technology), anti-AKT (1:1,000, Cell Signaling Technology), anti-P-GSK3β (1:1,000, Cell Signaling Technology), anti-GSK3β (1:1,000, Cell Signaling Technology), anti-FYN (1:1,000), anti-NRF2 (1:1,000), anti-BAX (1:1,000, Cell Signaling Technology), anti-BCL-2 (1:1,000, Abcam), anti-CLEAVED CASPASE-3 (C-CASPASE 3, 1:1,000, Cell Signaling Technology), anti-GAPDH (1:1,000, Servicebio technology), and anti-β-ACTIN (1:2000, Servicebio technology). The catalogs of antibodies are shown in Supplementary Table S1.

### Statistical Analysis

Data were analyzed by GraphPad Prism using one-way analysis of variance (ANOVA) or two-way ANOVA with Tukey’s or Sidak’s post hoc test as appropriate and presented as means ± standard deviation (SD). *p* < 0.05 was accepted significant difference among compared groups.

## Results

### RES Reversed HG-Induced Inhibition of Cell Viability and Apoptosis in MC3T3-E1 Cells

**FIGURE 1:**
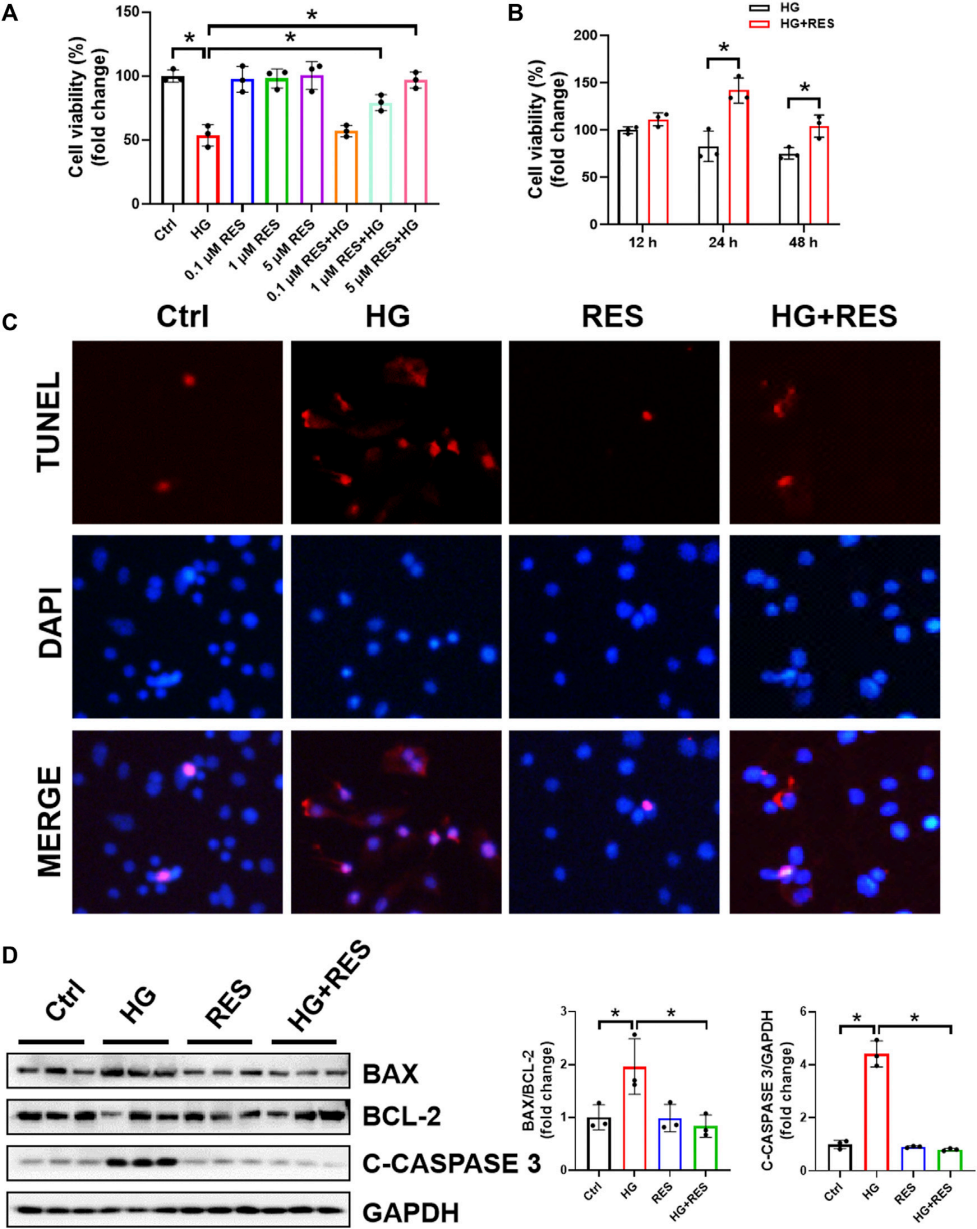
Resveratrol (RES) reversed high glucose- (HG-) induced inhibition of cell viability and apoptosis in MC3T3-E1 cells. **(A)** MC3T3-E1 cells were incubated with or without HG (30 mM) and RES (0.1, 1, and 5 μM) for 24 h. Cell Counting Kit 8 was used to measure cell viability. Three independent experiments were performed. **(B)** Cells were treated with HG or HG combined with RES (5 μM) for 12, 24, or 48 h. Cell viability was detected by Cell Counting Kit 8. Three independent experiments were performed. **(C)** Apoptotic cells, cultured in a basic culture medium in the presence or absence of 30 mM HG and (or) 5 μM RES for 24 h, were detected by TUNEL staining. DAPI staining was used to show the location of nuclei. **(D)** The expression of BAX, BCL-2, and CLEAVED-CASPASE 3 (C-CASPASE 3) was analyzed by Western blot with densitometric quantification. GAPDH as an internal control. Three independent experiments were performed. Data are shown as mean ± SD. *p < 0.05.

To ascertain the effect of RES on MC3T3-E1 cells under a diabetic condition, we first assessed the cell viability by CCK8 assay with different concentrations of RES. As shown in Figure 1A, cell viability was decreased after HG treatment, but RES ameliorated HG-inhibited cell proliferation in a dose-dependent manner. Figure 1B also indicated that this damage was significantly ameliorated by 5 μM RES for 24 h. Hence, 5 μM RES for 24 h was selected in the following experimental design. Given that apoptosis is a major contributor to HG-triggered osteoblast dysfunction, apoptotic cells were determined by TUNEL staining. Results showed that TUNEL-positive cells induced by HG could be reduced following RES treatment (Figure 1C). The inhibitory effects of RES on cell apoptosis were further confirmed by detecting apoptosis-related proteins, including BAX, BCL-2, and C-CASPASE3. Results of Western blot analysis showed that RES decreased BAX/BCL-2 ratio and C-CASPASE3 expression in HG-treated MC3T3-E1 cells (Figure 1D). The above findings implied that RES possessed an effective anti-apoptotic property under the HG condition.

### The Protection of RES on HG-Suppressed Osteogenic Differentiation

**FIGURE 2:**
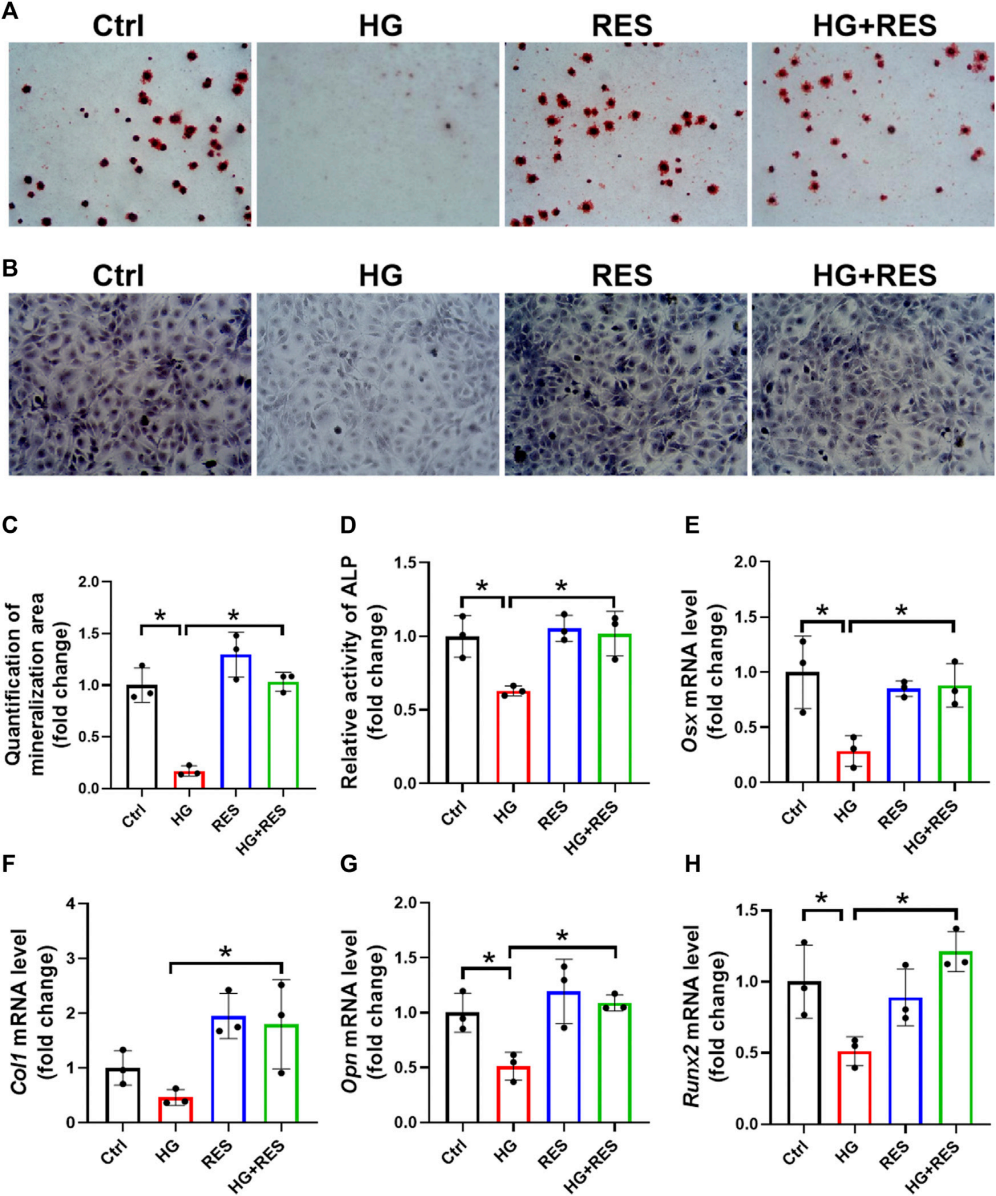
Protection of RES on HG-suppressed osteogenic differentiation. **(A–D)** MC3T3-E1 cells were treated in an osteogenic differentiation medium in the presence or absence of 30 mM HG and (or) 5 μM RES for 14 days. **(A,C)** Representative images of alizarin red staining and qualification of mineralization area for different groups. Three independent experiments were performed. **(B)** Representative images of alkaline phosphatase (ALP) staining. **(D)** Detection of ALP activity. Three independent experiments were performed. **(E–H)** mRNA levels of osterix (*Osx*), collagen 1 (*Col1*), osteopontin (*Opn*), and runt-related transcription factor 2 (*Runx2*) were detected by RT-qPCR. Three independent experiments were performed. Data are shown as mean ± SD. *p < 0.05.

MC3T3-E1 cells were cultured in the osteogenic differentiation medium for 14 days to test the effect of RES on osteogenic differentiation. The osteogenic differentiation ability was measured by ARS, which detected the mineralizing matrix. The HG-inhibited capability of osteogenic differentiation was significantly rescued by RES, as shown in Figures 2A,C. ALP, another biomarker of osteogenic differentiation, was used to evaluate the differentiation potential of preosteoblasts in the HG environment. Figures 2B,D demonstrated that the impaired process of osteogenic differentiation following the HG treatment was improved by RES. Consistent with the above data, mRNA expression of osteoblast-related genes (*Osx*, *Col1*, *Opn*, and *Runx2*) were upregulated by RES in HG-treated MC3T3-E1 cells (Figures 2E–H). Collectively, these findings suggested that RES could promote osteogenesis in MC3T3-E1 cells under the HG condition.

### The Protective Effect of RES on HG-Induced Oxidative Stress in MC3T3-E1 Cells

**FIGURE 3:**
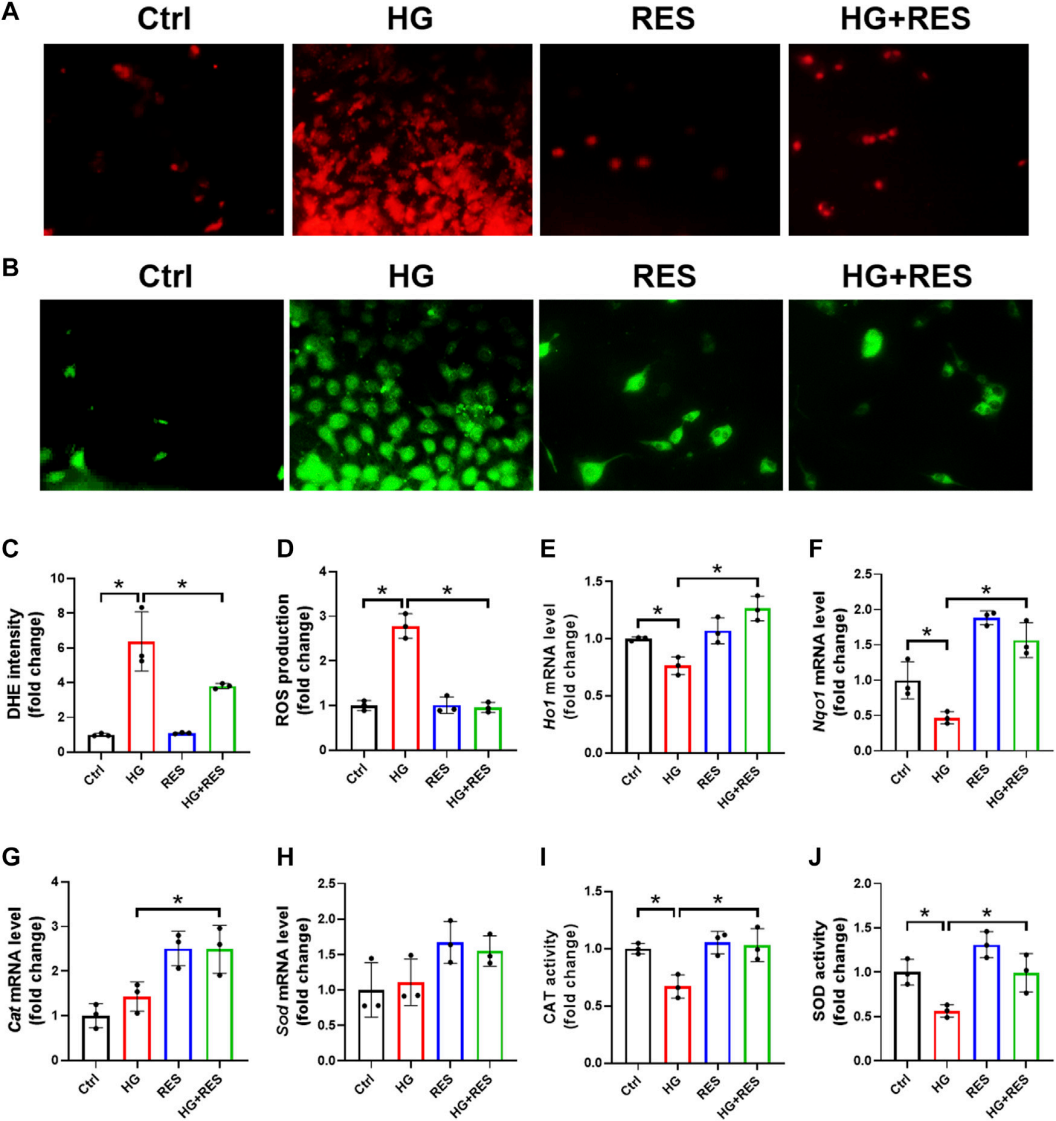
Protective effect of RES on HG-induced oxidative stress in MC3T3-E1 cells. MC3T3-E1 cells were incubated in a basic culture medium, together with or without HG (30 mM) and RES (5 μM) for 24 h. **(A–D)** Representative images of reactive oxygen species (ROS) levels determined by **(A,C)** DHE and **(B,D)** DCFH-DA staining with quantifications of the respective fluorescence intensity. Three independent experiments were performed. **(E–H)** Heme oxygenase 1 (*Ho1*), NAD(P)H quinone dehydrogenase 1 (*Nqo1*), catalase (*Cat*), and superoxide dismutase (*Sod*) mRNA levels were detected by RT-qPCR. Three independent experiments were performed. **(I,J)** Detection of CAT and SOD activities. Three independent experiments were performed. Data are shown as mean ± SD. *p < 0.05.

Oxidative stress is a major factor responsible for osteoblast dysfunction. Herein, we confirmed the effect of RES on HG-induced oxidative damage by DHE staining. As shown in Figures 3A,C, excessive ROS production induced by HG was largely reduced by RES treatment. Consistent with the DHE data, RES significantly reduced oxidative stress levels increased by HG as measured by DCFH-DA staining (Figures 3B,D). Moreover, relative mRNA levels of antioxidant genes (*Ho1* and *Nqo1*) were detected by RT-qRCR. Significant increases in their expression were shown in the HG combined with RES group compared with the HG group (Figures 3E,F). Although the mRNA levels of *Cat* and *Sod* had no apparent change in the HG group, HG treatment could significantly decrease the enzyme activities of CAT and SOD, which were upregulated following RES treatment (Figures 3G–J). Our findings indicated that RES acted as a strong candidate to reverse HG-induced oxidative stress.

### RES Protected From HG-Triggered Osteoblast Dysfunction *via* AKT/GSK3β/FYN-Mediated NRF2 Activation

**FIGURE 4:**
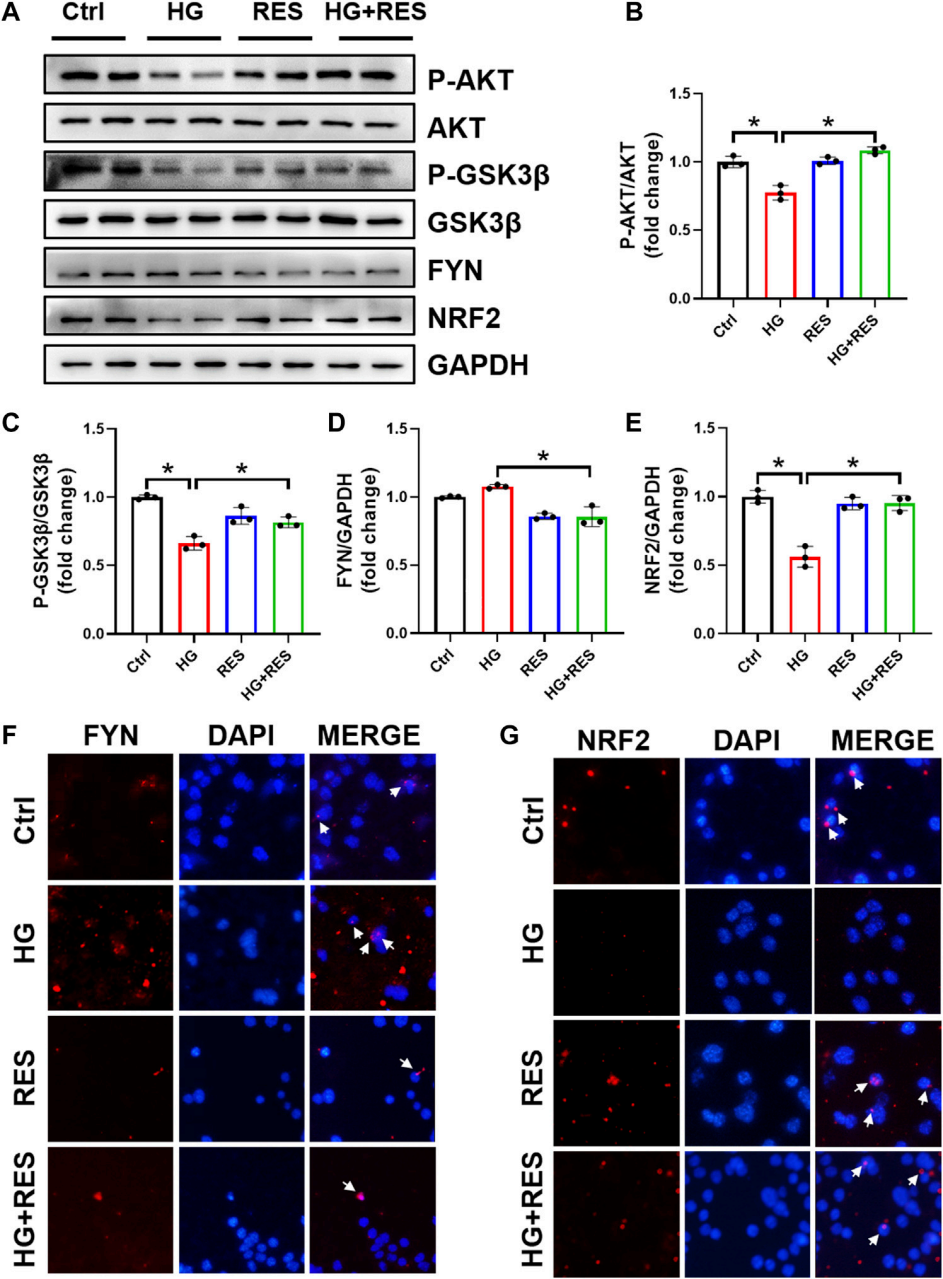
Alternations of AKT, glycogen synthase kinase 3β (GSK3β), FYN, and nuclear factor erythroid 2-related factor 2 (NRF2) protein expression under the HG condition with RES treatment in MC3T3-E1 cells. MC3T3-E1 cells were treated with vehicle, 30 mM HG alone, 5 μM RES alone, or HG and RES for 24 h. **(A–E)** The protein expression of P-AKT, AKT, P-GSK3β, GSK3β, FYN, and NRF2 was detected by Western blot. GAPDH as an internal control. Three independent experiments were performed. **(F,G)** Representative images of immunofluorescent staining for **(F)** FYN and **(G)** NRF2 nuclear accumulation. Data are shown as mean ± SD. *p < 0.05.

**FIGURE 5:**
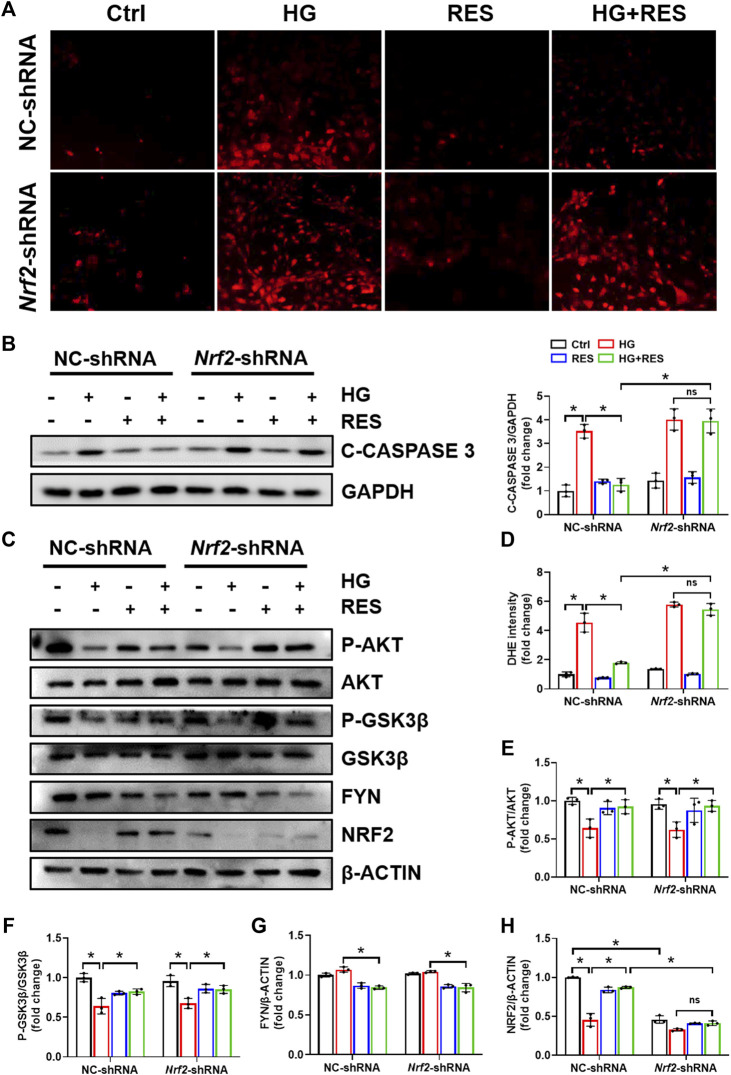
Knockdown of *Nrf2* eliminated the protective effect of RES on MC3T3-E1 cells injured by HG. Cells were transfected with NC-shRNA or *Nrf2*-shRNA, followed by HG (30 mM), RES (5 μM), neither, or both for 24 h. **(A,D)** Representative images of DHE staining and quantitative analysis of fluorescence intensity in MC3T3-E1 cells. Three independent experiments were performed. **(B)** C-CASPASE 3 expression was analyzed by Western blot followed by densitometric quantification. GAPDH as an internal control. Three independent experiments were performed. **(C,E–H)** Western blot analysis of P-AKT, AKT, P-GSK3β, GSK3β, FYN, and NRF2 expression levels and densitometric quantification of related proteins in MC3T3-E1 cells. β-ACTIN as an internal control. Three independent experiments were performed. Data are shown as mean ± SD. *p < 0.05; ns indicates no significance.

NRF2 plays a critical role in the cellular response to oxidative stress. When treated with HG, total and nuclear NRF2 expression significantly declined in MC3T3-E1 cells, supported by Western blot and nuclear immunofluorescent localization, which were obviously reversed by RES (Figures 4A,E,G). Moreover, several mRNA levels of NRF2-targeted genes, *Ho1* and *Nqo1*, were reduced in the HG condition, but they were completely recovered after RES treatment (Figures 3E,F), which suggested that RES could activate NRF2 transcriptional function in MC3T3-E1 cells under HG condition. To further investigate the effect of NRF2 in the protection of RES against HG-induced osteoblast damage, the *Nrf2* gene was knocked down by *Nrf2*-shRNA. Results of fluorescent probe DHE and Western blot indicated that knockdown of *Nrf2* blocked the protective effect of RES on oxidative stress and apoptosis (Figures 5A,B,D).

**FIGURE 6:**
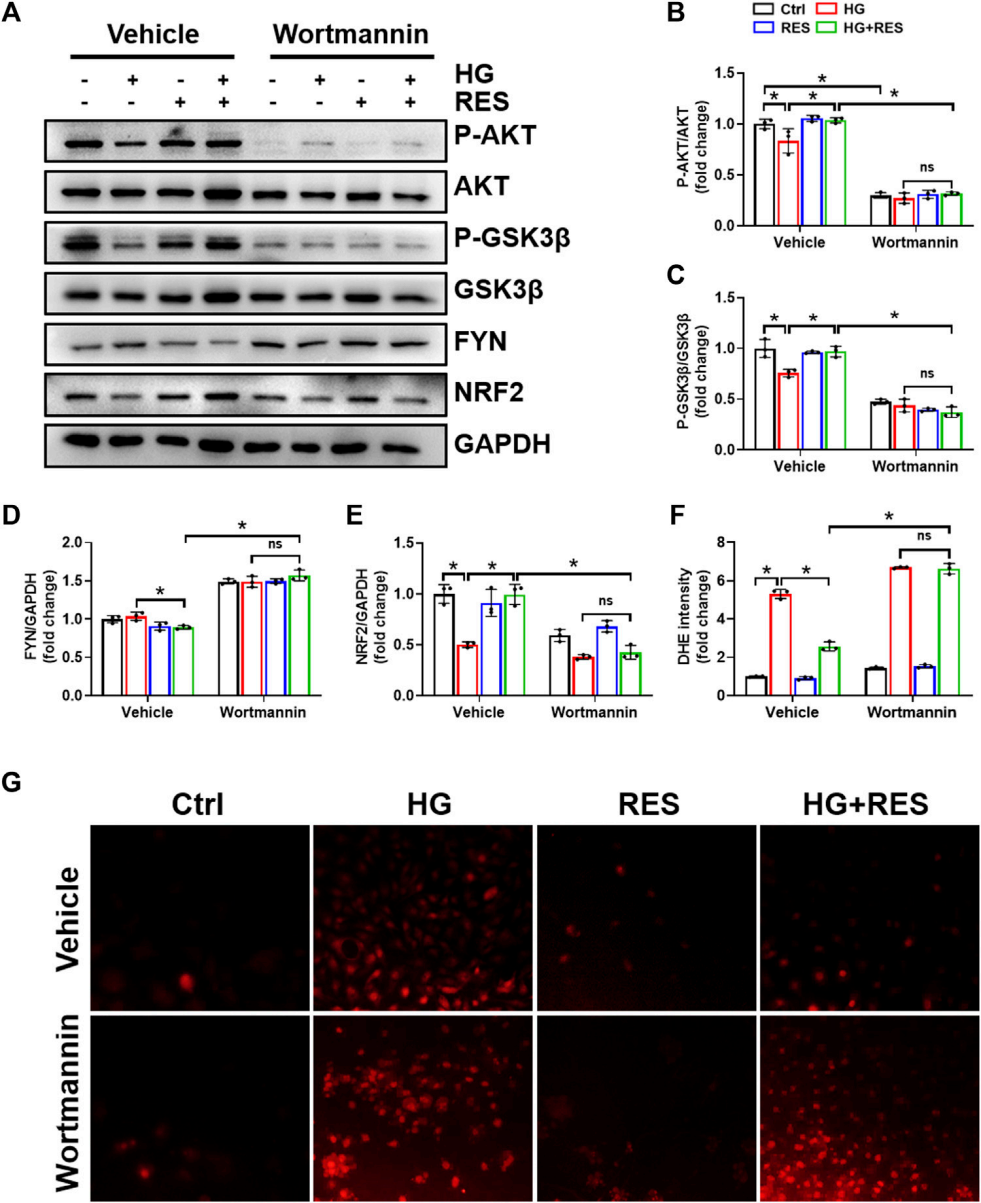
Wortmannin abolished the protection of RES on HG-induced osteoblast dysfunction. MC3T3-E1 cells were pretreated with wortmannin (10 μM) for 6 h and then exposed to HG (30 mM) in the presence or absence of RES (5 μM) for 24 h. **(A–E)** The protein expression of P-AKT, AKT, P-GSK3β, GSK3β, FYN, and NRF2 was evaluated by Western blot with densitometric quantification. GAPDH as an internal control. Three independent experiments were performed. **(F,G)** ROS production was determined with the fluorescent indicator DHE and measured by a fluorescence microscope. Three independent experiments were performed. Data are shown as mean ± SD. *p < 0.05; ns indicates no significance.

Previous studies indicated that the reduction of oxidative damage by RES was mainly associated with AKT-regulated NRF2 activation. Furthermore, the protective effect of NRF2 can be modulated by AKT/GSK3β/FYN. We found that exposing MC3T3-E1 cells to HG condition significantly decreased the expression of P-AKT and P-GSK3β and increased FYN expression in nuclei. RES reversed HG-induced changes in P-AKT, P-GSK3β, and nuclear FYN protein expression (Figures 4A–D,F). In order to figure out whether the PI3K/AKT pathway was critical to all protective effects of RES under a diabetic condition, wortmannin, a specific pharmacological inhibitor of the PI3K/AKT pathway, was applied to test parameters in the following experiments. From Figures 6A–E, the protein expression of P-AKT, P-GSK3β, and NRF2 significantly decreased, while that of FYN increased following wortmannin treatment. Most importantly, decreased protein expression of P-AKT, P-GSK3β, and NRF2 and increased protein expression of FYN in wortmannin groups following HG treatment could not be reversed by RES (Figures 6A–E). Moreover, DHE staining showed that the anti-oxidative ability of RES was largely abolished by wortmannin (Figures 6F,G). The mechanism of RES in regulating NRF2 was further verified by the knockdown of the *Nrf2* gene. As shown in Figure 5C, E–H, downregulation of the NRF2 expression did not affect the expression of P-AKT, P-GSK3β, and FYN in the *Nrf2*-shRNA groups compared with NC-shRNA groups. These findings proved that RES largely restrained HG-increased oxidative stress levels and prevented osteoblast dysfunction under the HG condition by activating NRF2 *via* AKT/GSK3β/FYN pathway.

## Discussion

Osteoblast dysfunction plays a crucial role in developing osteoporosis (Baek et al., 2010). However, there is no sufficient research on its pathology and promising therapeutic strategies. Our study focused on the protective function and molecular mechanism of RES in treating osteoblast dysfunction induced by HG. We have discovered for the first time that RES efficaciously alleviated HG-induced osteoblast dysfunction. Moreover, the present study results revealed that the protection of RES in improving HG-induced osteoblast dysfunction required NRF2 nuclear accumulation and transcriptional function. Most importantly, AKT/GSK3β/FYN pathway mediated the positive effect of RES on NRF2 activation.

The first novel finding of our study was that RES exerted a protective role in osteoblast dysfunction triggered by HG. In the present study, we found that HG indeed impaired cell viability and the differentiation ability of osteoblasts and promoted cell apoptosis. However, these adverse effects were reversed obviously following RES treatment (Figures 1, 2). Reportedly, oxidative stress can not only induce cell apoptosis and inhibit osteoblast differentiation but also play a critical role in the occurrence and progression of diabetic complications (Kitada et al., 2011; Han B. et al., 2019; Li et al., 2020). Consistent with previous studies, ROS levels significantly increased in MC3T3-E1 cells under the HG condition, which could be reversed by RES (Figure 3). These results indicated that RES strongly protected MC3T3-E1 cells against HG-induced oxidative damage and acted as a powerful antioxidant to suppress ROS production. Our findings presented that RES was a strong candidate for correcting the impairment of osteoblast function under the diabetic condition. However, the exact molecular mechanism for the protection of RES against osteoblast dysfunction caused by HG remained unknown.

Another important finding of our work was that the benefit of RES required activation of NRF2 antioxidant signaling. NRF2 is a master redox regulator by binding to regulatory antioxidant response elements to control ROS production (Harris and DeNicola 2020). Lin et al. found that RES promoted translocation of NRF2 from the cytoplasm into the nucleus and further initiated its downstream antioxidant genes *via* Kelch-like ECH-associated protein 1 (Lin et al., 2015). Upregulation of NRF2 and its downstream factor, HO-1, has already been testified to suppress dexamethasone-triggered oxidative stress of osteoblasts (Han D et al., 2019). This gave an idea that RES may protect osteoblasts against oxidative stress through stimulation of NRF2. As expected, we found that RES abolished HG-decreased NRF2 expression and nuclear translocation of NRF2 (Figures 4A,E,G). Recent studies demonstrated that AKT/GSK3β/FYN pathway played a critical function in the regulation of NRF2 activation and degradation (Xin et al., 2018; Zhang et al., 2020). Consistent with previous studies, RES increased phosphorylation of AKT and inhibited GSK3β activity, which decreased nuclear accumulation of FYN (Figures 4A–D,F). Importantly, we revealed that wortmannin almost completely reversed RES-induced activation of NRF2 (Figures 6A,E). However, *Nrf2*-shRNA had no effect on HG-downregulated AKT and GSK3β phosphorylation levels and upregulated FYN protein expression (Figures 5C,E–H). Additionally, both wortmannin and *Nrf2*-shRNA completely eliminated the protective role of RES on oxidative stress (Figures 5A,D and Figures 6F,G). All results above proved that the protective role of RES for osteoblast dysfunction triggered by HG could be regulated by AKT/GSK3β/FYN pathway to stimulate NRF2 activation.

**FIGURE 7:**
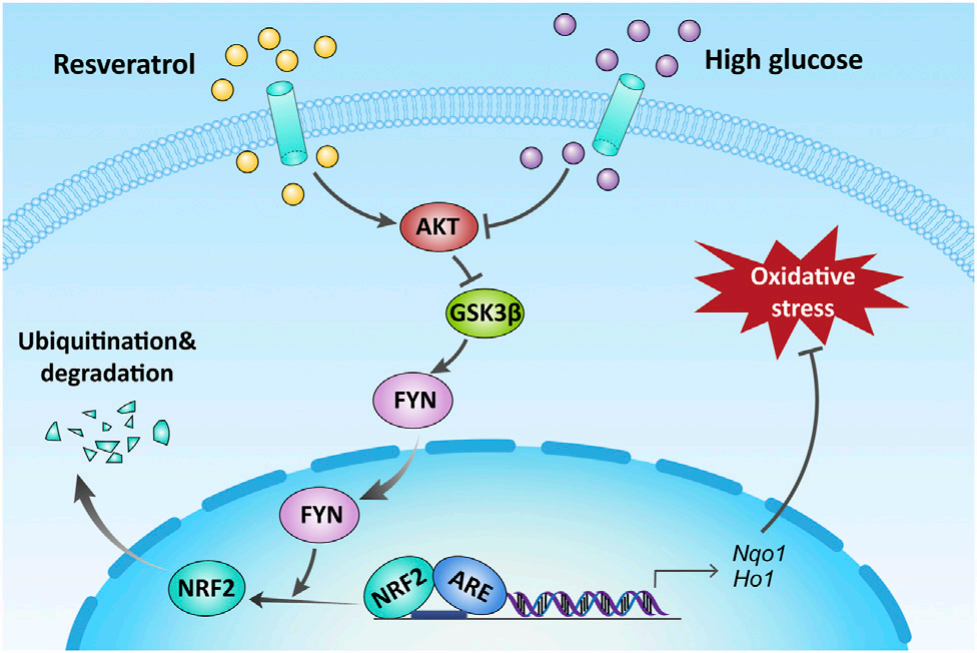
Schematic illustration of the protection of RES against HG-induced osteoblast dysfunction *via* AKT/GSK3β/FYN-mediated NRF2 activation. HG impaired the function of osteoblasts *via* oxidative stress. Upregulation of NRF2 by RES attenuated oxidative stress through promoting phosphorylation of AKT and GSK3β and inhibiting FYN under a diabetic condition.

In conclusion, the present work has provided sufficient evidence that RES could effectively ameliorate osteoblast dysfunction under the diabetic condition. This is predominantly owing to increased nuclear NRF2 expression, mediated by activating AKT, and inhibiting GSK3β and FYN-mediated NRF2 nuclear export and degradation in MC3T3 cells. Keeping NRF2 nuclear localization could activate NRF2 transcriptional function and upregulate the expression of antioxidant genes, protecting against HG-induced oxidative damage, as illustrated in Figure 7.

## Data Availability

The raw data supporting the conclusions of this article will be made available by the authors without undue reservation.
