# Supplementary material for: Resveratrol Attenuates High Glucose-Induced Osteoblast Dysfunction via AKT/GSK3β/FYN-Mediated NRF2 Activation
Source: Front Pharmacol. 2022 May 23;13:862618. doi: 10.3389/fphar.2022.862618 (PMC9169221; doi:10.3389/fphar.2022.862618)
Supplement: Supplementary file 1 [file Table1.DOCX]

Supplementary Material

| Antibody | Source | Catalog |
| --- | --- | --- |
| anti-P-AKT | Cell Signaling Technology | cat# 4060 |
| anti-AKT | Cell Signaling Technology | cat# 9272 |
| anti-P-GSK3β | Cell Signaling Technology | cat# 9336 |
| anti-GSK3β | Cell Signaling Technology | cat# 9315 |
| anti-FYN | Cell Signaling Technology | cat# 4023 |
| anti-NRF2 | Abcam | cat# ab31163 |
| anti-BAX | Cell Signaling Technology | cat# 2772 |
| anti-BCL-2 | Abcam | cat# ab182858 |
| anti-CLEAVED-CASPASE 3 | Cell Signaling Technology | cat# 9664 |
| anti-GAPDH | Servicebio technology | cat# GB11002 |
| anti-β-ACTIN | Servicebio technology | cat# GB11001 |

Table. S1 Catalogs of antibodies for western blot.
